# MALDI-TOF MS Distinctly Differentiates Nontypable *Haemophilus influenzae* from *Haemophilus haemolyticus*

**DOI:** 10.1371/journal.pone.0056139

**Published:** 2013-02-14

**Authors:** Bingqing Zhu, Di Xiao, Huifang Zhang, Yongchan Zhang, Yuan Gao, Li Xu, Jing Lv, Yingtong Wang, Jianzhong Zhang, Zhujun Shao

**Affiliations:** 1 State Key Laboratory for Infectious Disease Prevention and Control, National Institute for Communicable Disease Control and Prevention, Chinese Center for Disease Control and Prevention, Beijing, People’s Republic of China; 2 Hubei Provincial Center for Disease Control and Prevention, Wuhan, People’s Republic of China; 3 Hebei Provincial Center for Disease Control and Prevention, Shijiazhuang, People’s Republic of China; Universidade Federal do Rio de Janeiro, Brazil

## Abstract

Nontypable *Haemophilus influenzae* (NTHi) and *Haemophilus haemolyticus* exhibit different pathogenicities, but to date, there remains no definitive and reliable strategy for differentiating these strains. In this study, we evaluated matrix-assisted laser desorption/ionization time-of-flight mass spectrometry (MALDI-TOF MS) as a potential method for differentiating NTHi and *H. haemolyticus*. The phylogenetic analysis of concatenated 16S rRNA and recombinase A (*recA*) gene sequences, outer membrane protein P6 gene sequencing and single-gene PCR were used as reference methods. The original reference database (ORD, provided with the Biotyper software) and new reference database (NRD, extended with Chinese strains) were compared for the evaluation of MALDI-TOF MS. Through a search of the ORD, 76.9% of the NTHi (40/52) and none of the *H. haemolyticus* (0/20) strains were identified at the species level. However, all NTHi and *H. haemolyticus* strains used for identification were accurately recognized at the species level when searching the NRD. From the dendrogram clustering of the main spectra projections, the Chinese and foreign *H. influenzae* reference strains were categorized into two distinct groups, and *H. influenzae* and *H. haemolyticus* were also separated into two categories. Compared to the existing methods, MALDI-TOF MS has the advantage of integrating high throughput, accuracy and speed. In conclusion, MALDI-TOF MS is an excellent method for differentiating NTHi and *H. haemolyticus*. This method can be recommended for use in appropriately equipped laboratories.

## Introduction

Nontypable *Haemophilus influenzae* (NTHi) and *Haemophilus haemolyticus* are both commensal organisms that colonize the pharyngeal cavity [1]. NTHi is a particularly important pathogen, causing community-acquired pneumonia, chronic obstructive pulmonary disease and bronchiectasis exacerbations [2]–[4]. NTHi also causes meningitis, sinusitis and otitis media [4]–[6]. Comparatively, *H. haemolyticus* rarely causes invasive or surface infections [7]. However, in spite of the significant differences in the pathogenicity of these two species, there is no definitive and reliable strategy with which to differentiate these strains. For example, *H. haemolyticus* cannot be differentiated from NTHi by the production of ß-hemolysis on horse or rabbit blood agar because a significant portion of *H. haemolyticus* isolates appear nonhemolytic, similar to NTHi [8]; thus, this recommended standard microbiologic protocol is not sufficient.

Several molecular techniques have been used in attempts to differentiate the two species. Of these, PCR tests of single-gene targets, such as the lipo-oligosaccharide gene (*lgtC*), the IgA protease gene (*iga*), the fuculose kinase gene (*fucK*), the [Cu, Zn]-superoxide dismutase gene (*sodC*), the adherence and penetration protein gene (*hap*), and the outer membrane protein (*omp*) P2 and P6 gene, are the most simple and inexpensive [9]–[13]. However, a recent evaluation of several of these biomarkers revealed that none could unequivocally differentiate all NTHi isolates from *H. haemolyticus* [10]. Wang et al. exploited two probe-based real time PCR assays (*hpd#1* and *hpd#3*) targeting the protein D gene (*hpd*) and demonstrated satisfactory sensitivity in detecting 102 initial NTHi isolates (96% and 98%) [13], whereas in subsequent studies, Theodore et al. [14] and Binks et al. [10] observed lower sensitivity values for the same assays (88.5% and 88.9%, respectively). Multilocus sequence typing (MLST) is also used to segregate the two species, and it provides more accurate differentiation, but it is expensive and labor intensive [8]. In one study by Murphy et al., NTHi was differentiated from *H. haemolyticus* based on four specific amino acid residues in the conserved sequence of the P6 gene. Additionally, the monoclonal antibody 7F3, which recognizes amino acids 59 and 61, could also recognize specific amino acid residues of the P6 protein [8]. Subsequently, Chang et al. found that some NTHi strains could not be discriminated by this sequencing method [15].

Recently, as an alternative to biochemical and genome-based identification schemes, proteomic profiling by matrix-assisted laser desorption/ionization time-of-flight mass spectrometry (MALDI-TOF MS) has been successfully used in the species differentiation of a variety of microorganisms [16]–[20]. and been indicated that the potential to replace conventional identification techniques based on genomic fingerprinting [21] and biochemical methods [22]. The MALDI-TOF MS method can be performed rapidly and is reproducible using species-specific spectral patterns at a wide range of age of the culture, growth conditions, or medium selection [23]–[25]. We hypothesized that the NTHi and *H. haemolyticus* strains would have unique protein mass spectra despite their similarities in certain genes and proteins.

In this study, we evaluated MALDI-TOF MS coupled to the Biotyper system (Bruker Daltonics GmbH, Germany) as a potential method for discriminating NTHi and *H. haemolyticus*. The phylogenetic analysis of concatenated 16S rRNA and recombinase A (*recA*) gene sequences, P6 gene sequencing and single-gene PCR were used as the reference methods.

## Materials and Methods

### Identification of the Study Isolates

Fifty-two NTHi and 20 *H. haemolyticus* strains, in which 47 NTHi strains were isolated from the pharyngeal swabs of patients with upper respiratory infection and others were from asymptomatic carriers, were used in this study. These isolates were identified by colony morphology and dependence on X and V growth factors. All of the isolates were tested with polyvalent and monovalent antisera (2 ml; Remel Europe Ltd., Kent, United Kingdom), and no agglutination was observed. ß-hemolysis was evaluated by culturing strains on rabbit blood agar which contained heart infusion dehydrated medium and 5% defibrinated rabbit blood.

For the identification of nonhemolytic isolates, several tests were conducted as follows: **(1)** **P6 gene sequencing.** The P6 gene was sequenced and translated into a predicted amino acid sequence for each isolate, as specified by the reference protocol [8]. Four key residues in the P6 sequence were compared to the reference strains. **(2) PCR of** ***fucK*** **and** ***hpd*** **genes.** One standard PCR assay targeting the *fucK* gene [26] and one real-time PCR assay (*hpd#3*) [13] were performed on these strains. **(3) The phylogenetic analysis of concatenated 16S rRNA and** ***recA*** **gene sequences.** The same primers were used to amplify and sequence the 16S rRNA gene (forward 5′-AGAGTTTGATCCTGGCTCAG-3′, reverse 5′-CCGTCAATTCMTTTGAGTTT-3′) [27]. If amplification using these primers failed, an alternative forward primer (5′-ATGGCTCAGATTGAACGCTGG-3′) was used. Each PCR product was cloned into a plasmid, and one positive colony was sequenced if direct sequencing of the PCR product gave more than one peak at a single location. The procedures for the amplification and direct sequencing of the *recA* gene were described previously [26]. The selection and concatenation of the 16S rRNA and *recA* gene sequences, sequence alignments and phylogenetic analysis were conducted as described by Binks et al. [10]. The reference sequence of NTHi PittGG (GenBank accession No. CP000672) was analyzed, and the phylogenetic tree was rooted by one *H. parainfluenzae* strain (SZ56). These results were taken into account in the definition of NTHi and *H. haemolyticus*. Isolates that clustered with NTHi reference strains in the phylogenetic analysis of concatenated 16S rRNA and *recA* genes and with NTHi in two of the other three tests, were defined as NTHi; the remaining isolates were defined as *H. haemolyticus*.

### Sample Preparation for MALDI-TOF MS

Each strain was recovered on commercial chocolate agar plates supplemented with X and V growth factors. After culture at 37°C in a CO_2_ incubator (5%) overnight, one typical colony was subcultured for 18 to 24 hours. The cultures were visually checked for purity, and the ethanol/formic acid preparatory extraction method was used to prepare samples for MALDI-TOF MS analysis [28]. Two spots were prepared for each sample. A 1 µl volume of each supernatant protein sample was dropped onto an MSP 96 target ground steel 600-µm sample target (Bruker Daltonics GmbH, Germany) and allowed to dry before adding 1 µl of α-cyano-4-hydroxycinnamic acid (CHCA) [saturated matrix solution in 50% acetonitrile and 2.5% trifluoroacetic acid].

### Instrumentation and Data Acquisition

The Microflex LT™ (Bruker Daltonics GmbH, Germany) mass spectrometer was used for data acquisition. *Escherichia coli* ATCC 8739 was used as an external reference to calibrate the equipment, with a peak assignment tolerance of 250 ppm. The Microflex LT™ was equipped with an N_2_ laser (λ = 377 nm). The software used for data acquisition was FlexControl™ (version 3.0, Bruker Daltonics GmbH, Germany). The parameters used were as follows: mass range, 2,000–20,000 Da; ion source 1, 20 kV; ion source 2, 18.5 kV; lens, 8.45 kV; pulsed ion extraction, 320 ns; and laser frequency, 20.0 Hz. Each spectrum contained 100 shots, and 500 shots were superimposed to generate the total spectrum.

### Construction of the MALDI-TOF MS Reference Database

The Biotyper database contains only 10 *H. influenzae* references (these spectra are provided in the Biotyper software) (ORD) and no *H. haemolyticus* reference. Thus, in this study, a reference database was constructed using the automated functionality of the Biotyper (version 2.0) software package. Twenty strictly defined NTHi and *H. haemolyticus* strains (10 of each species) were included in the construction of the Biotyper reference database. For each database entry, 20 individually measured mass spectra were imported into the software. After spectrum smoothing, baseline correction, and peak picking, the resulting peak lists were used by the program to calculate and store a main spectrum containing information on the average peak mass, average peak intensity, and frequency.

### Evaluation of the MALDI-TOF MS Reference Database

For microorganism identification, the raw spectra of the unknown bacteria were imported into Biotyper software and analyzed by a built-in main spectra projection (MSP) feature, which is a proprietary algorithm for spectral pattern matching that returns a logarithmic score of 0 (no similarity) to 3 (absolute identity). The peak lists of unknown bacteria were compared with each entry in the Biotyper database, which currently contains 4015 bacterial reference spectra (NRD), including the 20 newly created reference spectra, using the standard parameters of the pattern-matching algorithm.

Forty-two NTHi and 10 *H. haemolyticus* isolates were used to evaluate the ORD and NRD. Scores ≥2.300 were rated as having a high degree of credibility at the species level; scores ≥2.000 were considered to indicate identification at the species level; scores from 1.700–1.999 were considered to indicate identification at least at the genus level; and scores <1.700 were interpreted as providing no identification information.

The 20 NTHi and *H. haemolyticus* strains which were used to construct the Biotyper reference database were also tested by searching the ORD and NRD^−minus^. The NRD^−minus^ was the NRD containing 4014 reference spectra without the spectra of the tested strain.

### The MSP Dendrogram

To visualize the phylogenetic distance between NTHi and *H. haemolyticus* strains and the relationship between Chinese and foreign *H. influenzae* strains, MSP dendrogram clustering of the 30 reference strains (10 original *H. influenzae* strains which were provided in the Biotyper reference database, 10 Chinese *H. influenzae* isolates, and 10 Chinese *H. haemolyticus* strains) in the MSP database was carried out using the standard settings in Biotyper.

### Nucleotide Sequence Accession Numbers

The sequences identified in the present study have been deposited in the GenBank database and can be found under the following accession numbers: for P6 gene, KC332001 to KC332072; for *recA* gene, KC332073 to KC332144; for 16S rRNA gene, KC332145 to KC332216.

## Results

### Reference Database Construction

For each database entry, individually measured mass spectra of NTHi and *H. haemolyticus* were imported into MSP, which performs normalization, smoothing, baseline correction and peak picking and generates a list of the most significant peaks. The program then calculates a major spectrum that contains the average peak mass, the average peak intensity and frequency. Twenty reference spectra were added to the ORD based on the newly created spectra of NTHi and *H. haemolyticus* strains that were isolated from China (Table 1). All of the newly entered NTHi strains were positive for the amplification of *fucK* and *hpd* genes.

**Table 1:** *H. influenzae* and *H. haemolyticus* strains added to the new reference database.

Species	Strains	Source of isolation	API NH code^a^	ß-hemolysis	*fucK*	*hpd*	*P6* b
*H. influenzae*	SC50438	Upper respiratory infection	7524	–	+	+	A,A,D,T
	SC41114	Upper respiratory infection	3624	–	+	+	A,A,D,T
	SC50249	Upper respiratory infection	1020	–	+	+	A,A,D,T
	SC40091	Upper respiratory infection	3424	–	+	+	A,A,D,T
	SC40619	Upper respiratory infection	7420	–	+	+	A,A,D,T
	SC40152	Upper respiratory infection	3224	–	+	+	A,A,D,T
	SC61124	Upper respiratory infection	1224	–	+	+	A,A,D,T
	SC60767	Upper respiratory infection	3420	–	+	+	A,A,D,T
	SC50579	Upper respiratory infection	3620	–	+	+	A,A,D,T
	SC1615	Upper respiratory infection	1624	–	+	+	A,A,D,T
*H. haemolyticus*	BD272-1	asymptomatic carriers	1024	–	–	–	G,S,N,E
	SZ14	asymptomatic carriers	1624	–	–	–	G,S,N,E
	SZ55	asymptomatic carriers	1424	–	–	–	G,S,N,E
	SZ45	asymptomatic carriers	3424	–	–	–	G,S,N,E
	SZ40	asymptomatic carriers	1624	–	–	–	G,S,N,E
	SZ22	asymptomatic carriers	3424	–	–	–	G,S,N,E
	SZ457	asymptomatic carriers	1624	+	–	–	G,S,N,E
	SZ41	asymptomatic carriers	1624	–	–	–	G,S,N,E
	BD262-1	asymptomatic carriers	1424	–	–	–	G,S,N,E
	BD346-b	asymptomatic carriers	1424	+	–	–	G,S,N,E

a The codes were the results of biochemical tests using API NH strips produced by bioMerieux Inc., Missouri USA.

b Amino acid residues in the P6 gene sequence at positions 33, 42, 59 and 61; A, A, D, T, Ala, Ala, Asp, Thr; G, S, N, E, Gly, Ser, Asn, Glu.

### Evaluation of the MALDI-TOF MS Reference Database

Among the 52 strains (42 NTHi and 10 *H. haemolyticus*) used to evaluate the MALDI-TOF MS reference database, the *fuc*K and *hpd* genes were amplified from 42 (100%) and 37 (88.1%) of the NTHi strains, respectively. All 10 *H. haemolyticus* strains were negative in the *fucK* and *hpd* assays, and specific key residues in the P6 sequences of all 52 strains were in accordance with those of the relative reference strains.

**Figure 1:**
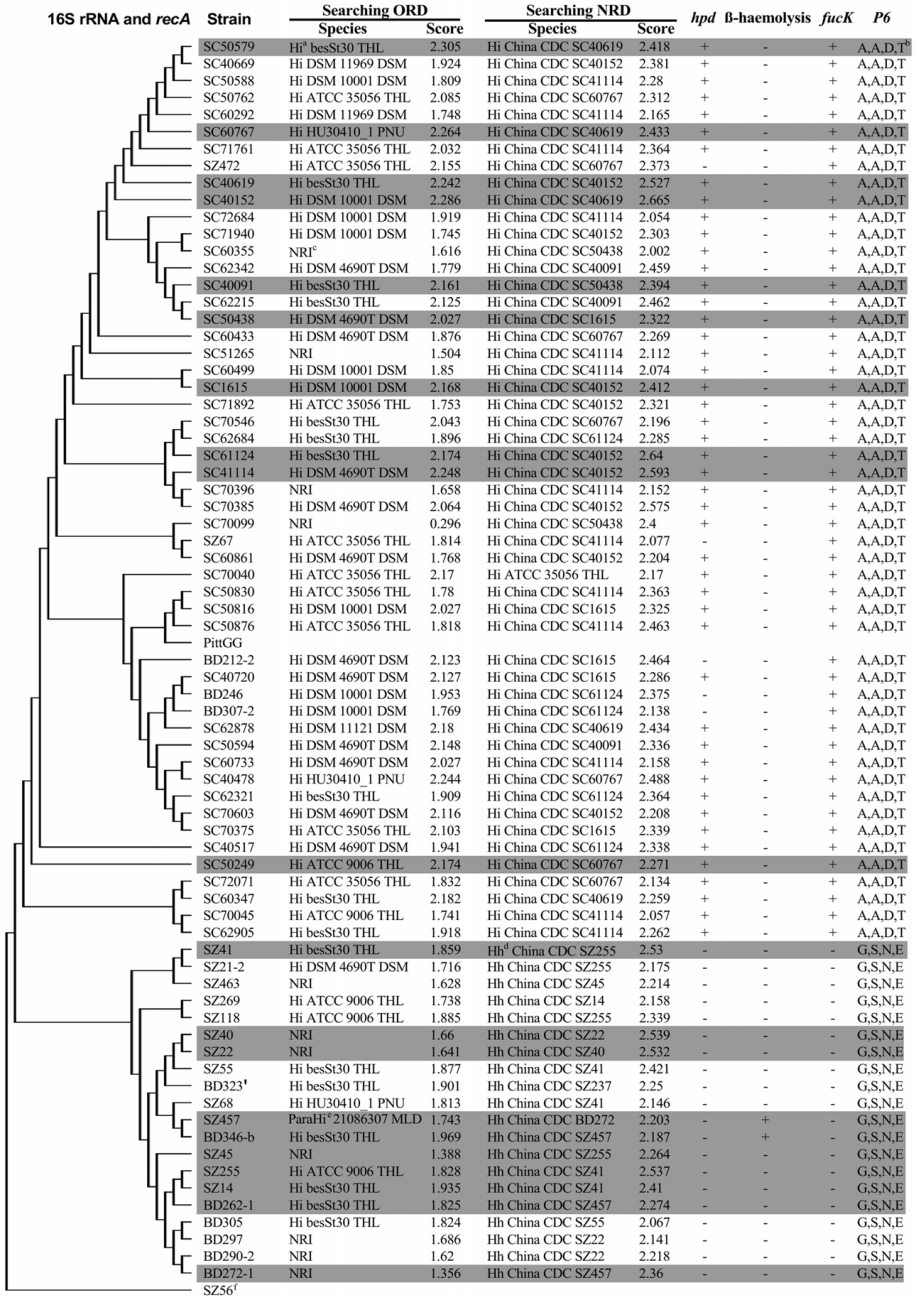
The comparison of the existing differentiation methods and the newly established MALDI-TOF MS method. The highlighted strains were used to construct the MALDI-TOF MS reference database. ^a^Hi, *H. influenza* ^b^Specific amino acid residues in the P6 gene sequence at positions 33, 42, 59 and 61 ^c^NRI, no reliable identification ^d^Hh, *H. haemolyticus* ^e^ParaHi, *H. parainfluenzae* ^f^ *H. parainfluenzae*, root of the phylogenetic tree.

By searching the ORD (3995 reference spectra), 38 (90.5%) and 30 (71.4%) of the 42 evaluation NTHi strains were identified at the genus level and species level, respectively; no strain was identified with a score greater than 2.3, while 4 strains were not identified. Comparatively, all 42 of the NTHi strains (100%) used to evaluate the MALDI-TOF MS reference database were identified at the species level by searching the NRD (4015 reference spectra). All NTHi strain first matched a reference spectrum from China in the database, with one exception, SC70040, matched H. influenzae ATCC 35056 THL. A total of 21 (50.0%) strains had scores higher than 2.3, which indicated a high degree of credibility at the species level. Five NTHi isolates that were *hpd* negative were identified as *H. influenzae*, all with scores higher than 2.0 (Figure 1).

For the identification of *H. haemolyticus*, 7 out of the 10 evaluation strains were recognized as *H. influenzae* with scores lower than 2.0 when referencing the ORD, and 3 were not identified. However, they were correctly identified at the species level by searching the NRD, and 2 (20%) of them were identified with scores greater than 2.3.

By searching the ORD, no *H. haemolyticus* and all NTHi strains which were used to construct the Biotyper reference database were correctly identified at the species level. But when searching the NRD^−minus^, all the 20 reference NTHi and *H. haemolyticus* strains were correctly identified. Fifteen of them (75%) had scores higher than 2.3.

A comparison of the existing differentiation methods and the newly established MALDI-TOF MS method is shown in Figure 1.

### The MSP Dendrogram

**Figure 2:**
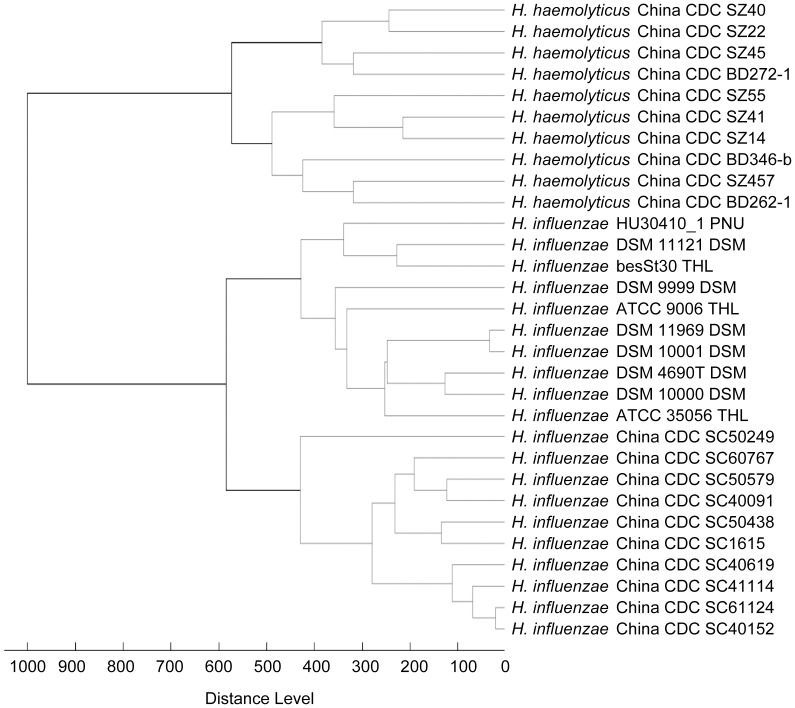
Cluster analysis of MALDI-TOF MS spectra of *H. influenzae* and *H. haemolyticus*.

A score-oriented MSP dendrogram was generated using the default settings in Biotyper 2.0 for the 30 *H. influenzae* and *H. haemolyticus* strains that were included in the MSP database. The NTHi and *H. haemolyticus* strains were categorized into two distinct groups. The *H. influenzae* strains from China and other foreign areas were also separated into two categories (Figure 2).

## Discussion

Because only 10 *H. influenzae* strains and no *H. haemolyticus* were included in the initial Biotyper 2.0 database, Chinese strains received low scores when compared to the reference strains. In this study, 20 strictly defined *H. influenzae* and *H. haemolyticus* strains were added to the database, enabling the accurate identification of the other Chinese strains. Therefore, an important and significant contribution of our study is that it extends and enriches the database for the identification of *H. influenzae* and *H. haemolyticus* strains.

The aim of this study was identifying a novel method for discriminating NTHi and *H. haemolyticus* strains. Although the NTHi and *H. haemolyticus* strains were separated into two distinct species by the phylogenetic analysis of concatenated 16S rRNA and *recA* gene sequences, which demonstrated a good distinguishing ability of this method, direct sequencing of the 16S rRNA PCR product did not provide unambiguous results for some strains. Alignment of the sequences of the 16S rRNA gene of reference NTHi strains shows that the six copies in one complete genome are not consistent. By MALDI-TOF MS, NTHi and *H. haemolyticus* were unambiguously distinguished from each other. The two species expressed distinct spectra in the MSP dendrogram, which showed that MALDI-TOF MS was a reliable method for discriminating NTHi and *H. haemolyticus*. Additionally, this detection method is suitable for high-throughput application and is very rapid compared to the phylogenetic analysis of 16S rRNA and *recA* gene sequences. In the definition of NTHi and *H. haemolyticus* strains, three other methods were also applied to ensure the accuracy. Although previous studies [10], [14] had shown less than 100% sensitivity and specificity for these methods, the testing results were consistent with each other in this study, except for the *hpd*#3 assay. This result guarantees the reliability of the definition of NTHi and *H. haemolyticus*, but it also suggests that the newly extended reference database should be evaluated with strains that were negative by other identification methods.

In the MSP dendrogram, *H. influenzae* strains from China and other areas were classified into two distinct categories, which revealed the difference between the protein mass spectra of these two panels of strains. This finding may explain why the majority of Chinese strains were only compared to those from China. Additionally, this provides a theoretical foundation for establishing a region-specific MALDI-TOF MS reference database. However, considering that some *H. influenzae* reference strains in the ORD are serotypable, the difference between spectra of *H. influenzae* strains from different countries cannot reflect the true distinction between the *H. influenzae* strains of two regions.

Although the aim of this research was to establish a new method for differentiating NTHi and *H. haemolyticus* strains, we compared the unknown bacteria with all of the entries in the Biotyper database. Therefore, there is a chance that NTHi and *H. haemolyticus* strains could be confused with those of other genera or other *Haemophilus* species. In fact, one *H. haemolyticus* strain was recognized as *H. parainfluenzae* at the genus level by searching the ORD. Therefore, 10 *H. parahaemophilus* and 3 *H. parahaemolyticus* strains were also analyzed to assess whether MALDI-TOF MS was suitable to identify *H. influenzae* and *H. haemolyticus*. Encouragingly, all of these strains were accurately identified, which confirmed the ability of MALDI-TOF MS to identify NTHi and *H. haemolyticus* and also implied that this method has the potential to be used for the identification of other *Haemophilus* species.

In conclusion, MALDI-TOF MS was an excellent method for differentiating NTHi and *H. haemolyticus*. This method can be recommended in appropriately equipped laboratories.

## References

[1] Mukundan D, Ecevit Z, Patel M, Marrs CF, Gilsdorf JR (2007) Pharyngeal colonization dynamics of Haemophilus influenzae and Haemophilus haemolyticus in healthy adult carriers. J Clin Microbiol 45: 3207–3217. 17687018 10.1128/JCM.00492-07 PMC2045313

[2] Tao LL, Hu BJ, He LX, Wei L, Xie HM, et al (2012) Etiology and antimicrobial resistance of community-acquired pneumonia in adult patients in China. Chin Med J (Engl) 125: 2967–2972. 22932165

[3] Sethi S, Murphy TF (2001) Bacterial infection in chronic obstructive pulmonary disease in 2000: a state-of-the-art review. Clin Microbiol Rev 14: 336–363. 11292642 10.1128/CMR.14.2.336-363.2001 PMC88978

[4] Murphy TF (2003) Respiratory infections caused by non-typeable Haemophilus influenzae. Curr Opin Infect Dis 16: 129–134. 12734445 10.1097/00001432-200304000-00009

[5] Musser JM, Barenkamp SJ, Granoff DM, Selander RK (1986) Genetic relationships of serologically nontypable and serotype b strains of Haemophilus influenzae. Infect Immun 52: 183–191. 3485574 10.1128/iai.52.1.183-191.1986 PMC262217

[6] Pichichero ME, Casey JR, Hoberman A, Schwartz R (2008) Pathogens causing recurrent and difficult-to-treat acute otitis media, 2003–2006. Clin Pediatr (Phila) 47: 901–906. 18559884 10.1177/0009922808319966

[7] Anderson R, Wang X, Briere EC, Katz LS, Cohn AC, et al (2012) Haemophilus haemolyticus isolates causing clinical disease. J Clin Microbiol 50: 2462–2465. 22573587 10.1128/JCM.06575-11 PMC3405640

[8] Murphy TF, Brauer AL, Sethi S, Kilian M, Cai X, et al (2007) Haemophilus haemolyticus: a human respiratory tract commensal to be distinguished from Haemophilus influenzae. J Infect Dis 195: 81–89. 17152011 10.1086/509824

[9] McCrea KW, Xie J, LaCross N, Patel M, Mukundan D, et al (2008) Relationships of nontypeable Haemophilus influenzae strains to hemolytic and nonhemolytic Haemophilus haemolyticus strains. J Clin Microbiol 46: 406–416. 18039799 10.1128/JCM.01832-07 PMC2238123

[10] Binks MJ, Temple B, Kirkham LA, Wiertsema SP, Dunne EM, et al (2012) Molecular surveillance of true nontypeable Haemophilus influenzae: an evaluation of PCR screening assays. PLoS One 7: e34083. 22470516 10.1371/journal.pone.0034083 PMC3314702

[11] Norskov-Lauritsen N (2009) Detection of cryptic genospecies misidentified as Haemophilus influenzae in routine clinical samples by assessment of marker genes fucK, hap, and sodC. J Clin Microbiol 47: 2590–2592. 19535530 10.1128/JCM.00013-09 PMC2725679

[12] Abdeldaim GM, Stralin K, Kirsebom LA, Olcen P, Blomberg J, et al (2009) Detection of Haemophilus influenzae in respiratory secretions from pneumonia patients by quantitative real-time polymerase chain reaction. Diagn Microbiol Infect Dis 64: 366–373. 19446978 10.1016/j.diagmicrobio.2009.03.030

[13] Wang X, Mair R, Hatcher C, Theodore MJ, Edmond K, et al (2011) Detection of bacterial pathogens in Mongolia meningitis surveillance with a new real-time PCR assay to detect Haemophilus influenzae. Int J Med Microbiol 301: 303–309. 21276750 10.1016/j.ijmm.2010.11.004

[14] Theodore MJ, Anderson RD, Wang X, Katz LS, Vuong JT, et al (2012) Evaluation of new biomarker genes for differentiating Haemophilus influenzae from Haemophilus haemolyticus. J Clin Microbiol 50: 1422–1424. 22301020 10.1128/JCM.06702-11 PMC3318567

[15] Chang A, Adlowitz DG, Yellamatty E, Pichichero M (2010) Haemophilus influenzae outer membrane protein P6 molecular characterization may not differentiate all strains of H. influenzae from H. haemolyticus. J Clin Microbiol 48: 3756–3757. 20686092 10.1128/JCM.01255-10 PMC2953139

[16] De Carolis E, Posteraro B, Lass-Florl C, Vella A, Florio AR, et al (2011) Species identification of Aspergillus, Fusarium and Mucorales with direct surface analysis by matrix-assisted laser desorption ionization time-of-flight mass spectrometry. Clin Microbiol Infect 18: 475–484. 21883662 10.1111/j.1469-0691.2011.03599.x

[17] Murugaiyan J, Ahrholdt J, Kowbel V, Roesler U (2012) Establishment of a matrix-assisted laser desorption ionization time-of-flight mass spectrometry database for rapid identification of infectious achlorophyllous green micro-algae of the genus Prototheca. Clin Microbiol Infect 18: 461–467. 21781208 10.1111/j.1469-0691.2011.03593.x

[18] Yan Y, He Y, Maier T, Quinn C, Shi G, et al (2011) Improved identification of yeast species directly from positive blood culture media by combining Sepsityper specimen processing and Microflex analysis with the matrix-assisted laser desorption ionization Biotyper system. J Clin Microbiol 49: 2528–2532. 21543564 10.1128/JCM.00339-11 PMC3147835

[19] Ferreira L, Sanchez-Juanes F, Garcia-Fraile P, Rivas R, Mateos PF, et al (2011) MALDI-TOF mass spectrometry is a fast and reliable platform for identification and ecological studies of species from family Rhizobiaceae. PLoS One 6: e20223. 21655291 10.1371/journal.pone.0020223 PMC3105015

[20] Vanlaere E, Sergeant K, Dawyndt P, Kallow W, Erhard M, et al (2008) Matrix-assisted laser desorption ionisation-time-of of-flight mass spectrometry of intact cells allows rapid identification of Burkholderia cepacia complex. J Microbiol Methods 75: 279–286. 18627778 10.1016/j.mimet.2008.06.016

[21] Doan NT, Van Hoorde K, Cnockaert M, De Brandt E, Aerts M, et al (2012) Validation of MALDI-TOF MS for rapid classification and identification of lactic acid bacteria, with a focus on isolates from traditional fermented foods in Northern Vietnam. Lett Appl Microbiol 55: 265–273. 22774847 10.1111/j.1472-765X.2012.03287.x

[22] Martiny D, Dediste A, Debruyne L, Vlaes L, Haddou NB, et al (2011) Accuracy of the API Campy system, the Vitek 2 Neisseria-Haemophilus card and matrix-assisted laser desorption ionization time-of-flight mass spectrometry for the identification of Campylobacter and related organisms. Clin Microbiol Infect 17: 1001–1006. 20673261 10.1111/j.1469-0691.2010.03328.x

[23] Valentine N, Wunschel S, Wunschel D, Petersen C, Wahl K (2005) Effect of culture conditions on microorganism identification by matrix-assisted laser desorption ionization mass spectrometry. Appl Environ Microbiol 71: 58–64. 15640170 10.1128/AEM.71.1.58-64.2005 PMC544247

[24] Sogawa K, Watanabe M, Sato K, Segawa S, Ishii C, et al (2011) Use of the MALDI BioTyper system with MALDI-TOF mass spectrometry for rapid identification of microorganisms. Anal Bioanal Chem 400: 1905–1911. 21442367 10.1007/s00216-011-4877-7

[25] De Bruyne K, Slabbinck B, Waegeman W, Vauterin P, De Baets B, et al (2011) Bacterial species identification from MALDI-TOF mass spectra through data analysis and machine learning. Syst Appl Microbiol 34: 20–29. 21295428 10.1016/j.syapm.2010.11.003

[26] Meats E, Feil EJ, Stringer S, Cody AJ, Goldstein R, et al (2003) Characterization of encapsulated and noncapsulated Haemophilus influenzae and determination of phylogenetic relationships by multilocus sequence typing. J Clin Microbiol 41: 1623–1636. 12682154 10.1128/JCM.41.4.1623-1636.2003 PMC153921

[27] Webster G, Parkes RJ, Cragg BA, Newberry CJ, Weightman AJ, et al (2006) Prokaryotic community composition and biogeochemical processes in deep subseafloor sediments from the Peru Margin. FEMS Microbiol Ecol 58: 65–85. 16958909 10.1111/j.1574-6941.2006.00147.x

[28] Xiao D, Zhao F, Lv M, Zhang H, Zhang Y, et al (2012) Rapid identification of microorganisms isolated from throat swab specimens of community-acquired pneumonia patients by two MALDI-TOF MS systems. Diagn Microbiol Infect Dis 73: 301–307. 22633336 10.1016/j.diagmicrobio.2012.04.004

